# Vitamin D receptor as a marker of prognosis in oesophageal adenocarcinoma: a prospective cohort study

**DOI:** 10.18632/oncotarget.26151

**Published:** 2018-09-28

**Authors:** Stephen McCain, James Trainor, Damian T. McManus, Úna C. McMenamin, Stephen McQuaid, Victoria Bingham, Jacqueline A. James, Manuel Salto-Tellez, Richard C. Turkington, Helen G. Coleman

**Affiliations:** ^1^ Cancer Epidemiology Research Group, Centre for Public Health, Queen's University Belfast, Belfast, Northern Ireland; ^2^ Department of Pathology, Belfast Health and Social Care Trust, Belfast, Northern Ireland; ^3^ Centre for Cancer Research and Cell Biology, Queen's University Belfast, Belfast, Northern Ireland

**Keywords:** vitamin D receptor, oesophageal cancer, oesophageal adenocarcinoma

## Abstract

**Aims:**

Vitamin D receptor (VDR) expression has been associated with survival in several cancer sites. This study aims to evaluate the association between VDR expression and prognosis in oesophageal adenocarcinoma patients.

**Results:**

During a median of 2.5 (maximum 9) years of follow-up, 75 patients died. In analysis adjusted for confounders, higher VDR expression was associated with an improved overall survival (HR 0.49 95% CI 0.25–0.96) and disease-specific survival (HR 0.50 95% CI 0.26–0.99), when comparing the highest with the lowest tertile of expression. These associations were strongest in sensitivity analysis restricted to junctional tumours.

**Conclusions:**

This study is the first to demonstrate that patients with higher VDR expression in oesophageal adenocarcinoma have a more favourable prognosis. Further work is needed to validate these findings, and to define the role of VDR in the aetiology, progression and management of oesophageal adenocarcinoma.

**Methods:**

Oesophageal adenocarcinoma specimens and clinical data were collected from 130 patients treated with neo-adjuvant chemotherapy prior to surgical resection at the Northern Ireland Cancer Centre between 2004 and 2012. Tissue microarrays were created and immunohistochemical staining for VDR was performed on triplicate tumour cores from each resection specimen. Cox proportional hazards models were applied to evaluate associations between VDR, according to tertiles of expression, and survival outcomes.

## INTRODUCTION

Oesophageal cancer causes 400,000 deaths worldwide each year and ranks as the sixth most common cause of cancer mortality [1]. Neo-adjuvant therapy has somewhat improved prognosis, however 5-year survival rates for this malignancy still only range between 10% and 18% in Western settings [2, 3]. These low figures are partially related to more than 30% of oesophageal cancer patients having metastatic disease at first presentation [2]. Even in those patients with localised disease who have undergone attempted curative surgery, the 5-year survival is still as low as 41% [2]. In addition to early detection initiatives, there is a need to identify actionable, prognostic biomarkers to help predict patient outcome and also to identify novel therapeutic targets.

The vitamin D receptor (VDR) exerts its biological influence by binding with circulating vitamin D, and thereby contributes to the regulation of apoptosis and cell differentiation, and suppression of cancer cell proliferation [4–6]. *In-vitro* studies within colorectal cancer cell lines have demonstrated that cells with high VDR expression tend to be well differentiated and are biologically favourable, whereas cell lines with low VDR expression demonstrated aggressive features with higher metastatic potential [7]. These findings have been translated in clinical studies which have shown that high VDR expression has been associated with increased survival in colorectal, pancreatic and breast cancer, cutaneous melanoma, urothelial bladder cancer and oesophageal squamous cell carcinoma [8–13].

To date, there has been little research investigating VDR expression and oesophageal adenocarcinoma outcomes. However, several published papers have reported differences in VDR expression when comparing native, pre-malignant and oesophageal adenocarcinoma tissue in cross-sectional analyses from different patients [14, 15]. One study reported no VDR staining in normal oesophageal squamous mucosa, whereas Barrett's mucosa and low grade dysplasia had strongly positive VDR staining (95% and 100%, respectively), which then decreased slightly in tissue from patients with adenocarcinoma (79%) [14]. This study is discussed in depth in the discussion section. Similar findings were observed in a small study which assessed VDR expression in tumour, adjacent normal and Barrett's mucosa, from five oesophageal adenocarcinoma resection specimens [15]. Collectively, these findings suggest that VDR expression only features in oesophageal cells after they have undergone metaplastic transition, but it is unclear if this is a cause-or-effect role. The implications of VDR expression on further progression of columnar epithelium to oesophageal adenocarcinoma, and prognosis after adenocarcinoma development remains unclear.

To date, only one study has investigated the association between VDR expression and oesophageal adenocarcinoma outcomes in 116 patients. In this patient cohort from the University of Rochester, New York, no significant difference in outcome in those patients with high compared to low VDR expression was seen [14].

This study aims to expand on this limited evidence, to investigate the association between VDR expression and prognosis in oesophageal adenocarcinoma patients who have undergone neoadjuvant chemotherapy and surgical resection.

## RESULTS

### Patient demographics and tumour characteristics

Of the total 130 oesophageal adenocarcinoma patients in this study, 78% were male and 22% were female. The majority of tumours were located at the gastro-oesophageal junction (84.6%), with Siewert 1 tumours the most common (50.8%), followed by Siewert 2 (25.4%) and Siewert 3 (8.5%).

Table 1 presents the patient demographics and tumour characteristics across tertiles of maximum VDR expression. There was no difference by patient sex, age at diagnosis, year of diagnosis, smoking, or alcohol status according to tertiles of VDR expression. There were fewer Siewert 1 tumours in the highest compared with the lowest tertile of VDR expression (*p* = 0.04). There was also a difference in T-stage (*p* = 0.04) according to tertiles of VDR expression, although this mostly reflect small numbers in some categories. There was no difference in lymphovascular invasion, circumferential resection margin status, tumour differentiation or surgical nodal status according to tertiles of VDR expression.

**Table 1:** Patient demographics and tumour characteristics according to tertiles of maximum VDR expression

	Total number *n* = 130	Tertile 1 H score <120 *n* = 48	Tertile 2 H score 120–240 *n* = 47	Tertile 3 H score >240 *n* = 35	*P*-value
**Sex**					
Male	101 (77.7)	34 (70.8)	38 (80.9)	29 (82.9)	
Female	29 (22.3)	14 (29.2)	9 (19.2)	6 (17.1)	0.35
**Age at diagnosis (years)**					
<50	14 (10.8)	6 (12.5)	4 (8.5)	4 (11.4)	
50–59	25 (19.2)	7 (14.6)	11 (23.4)	7 (20.0)	0.79
60–79	61 (46.9)	26 (54.2)	21 (44.7)	14 (40.0)	
≥80	30 (23.1)	9 (18.8)	11 (23.4)	10 (28.6)	
**Smoking status**					
Never smoker	34 (26.2)	12 (25)	11 (23.4)	11 (31.4)	
Former smoker	48 (36.9)	18 (37.5)	17 (36.7)	13 (37.4)	
Current smoker	31 (23.9)	11 (22.9)	14 (29.8)	6 (17.1)	0.90
*Unknown*	17 (13.1)	7 (14.6)	5 (10.64)	5 (14.3)	
**Alcohol**					
Never drinker	41 (31.5)	12 (25.0)	15 (31.9)	14 (40.0)	
Ever	69 (53.1)	27 (56.3)	26 (55.3)	16 (45.7)	0.64
*Unknown*	20 (15.4)	9 (18.8)	6 (12.8)	5 (14.3)	
**Primary site**					
Lower third	20 (15.4)	6 (12.5)	6 (12.8)	8 (22.9)	
Gastro-oesophageal junction	110 (84.6)	42 (87.5)	41 (87.2)	27 (77.1)	0.36
Siewert classification					
1	66 (50.8)	24 (50)	31 (66)	11 (31.4)	
2	33 (25.4)	16 (33.3)	6 (12.8)	11 (31.4)	0.04
3	11 (8.5)	2 (4.2)	4 (8.5)	5 (14.3)	
**PET responder**					
No	43 (33.1)	16 (33.3)	14 (29.8)	13 (37.1)	
Yes	57 (43.9)	20 (41.7)	22 (46.8)	15 (42.9)	0.95
Unknown	32 (23.1)	12 (25.0)	11 (23.4)	7 (20.0)	
**Lymphatic vascular invasion**					
No	40 (30.8)	15 (31.3)	13 (27.7)	12 (34.3)	
Yes	90 (69.2)	33 (66.7)	34 (72.3)	23 (65.7)	0.81
**Circumferential resection margin status**					
Negative	73 (56.1)	26 (54.2)	27 (57.5)	20 (57.1)	
Positive	57 (43.9)	22 (45.8)	20 (42.6)	15 (42.9)	0.94
**Differentiation** Well or Moderate	52 (40.0)	22 (45.8)	17 (36.2)	13 (37.1)	
Moderate-Poor or Poor	78 (60.0)	26 (54.2)	30 (63.8)	22 (62.9)	0.58
**Surgical T stage**					
1	11 (8.5)	6 (12.5)	1 (2.1)	4 (11.4)	
2	25 (19.2)	4 (8.3)	15 (31.9)	6 (17.1)	
3	89 (68.5)	36 (75)	28 (59.6)	25 (71.4)	0.04
4	5 (3.9)	2 (4.2)	3 (6.4)	0 (0)	
**Surgical N stage**					
0	44 (33.9)	16 (33.3)	16 (34.0)	12 (34.3)	
1	27 (20.8)	11 (22.9)	10 (21.3)	6 (17.1)	
2	29 (22.3)	9 (18.8)	13 (27.7)	7 (20.0)	0.86
3	30 (23.1)	12 (25.0)	8 (17.0)	11 (28.6)	

Abbreviations: PET = Positive emission tomography scan; T-stage = Tumour stage; N stage = Nodal stage.

### Survival analysis

There were 75 patients who had died during a maximum of 9 (median 2.5) years of follow-up. As shown in Table 2, in unadjusted analysis, a higher VDR expression showed a trend towards significance with an improved survival. In adjusted analysis, a dose-response association between higher VDR expression and improved overall survival became apparent. In patients with tumour VDR expression in the middle tertile, there was a 40% non-significant reduced risk of death (HR 0.60 95% CI 0.33–1.09; *p* = 0.09) and those in the highest tertile had a 51% significantly lower risk of death (HR 0.49 95% CI 0.25–0.96; *p* = 0.04), compared with the lowest VDR expression category. This association was not as apparent in analysis evaluating high and low VDR expression as determined by the median cut-off; higher VDR expression was associated with an 18% non-significant reduced risk of death (HR 0.82 95% CI 0.48–1.38; *p* = 0.45) for high VDR expression compared with the low VDR expression group. Very similar patterns of results were observed in cancer-specific survival analysis (Table 2).

**Table 2:** Oesophageal adenocarcinoma survival outcomes according to Vitamin D receptor expression

	Dead *n* = 75	Alive *n* = 55	Unadjusted Hazard ratio (95% CI)	*P*-value	Adjusted Hazard ratio^a^ (95% CI)	*P*-value
*Overall survival*						
Low (<median)	39	23	1.00		1.00	
High (≥median)	36	32	0.71 (0.45–1.13)	0.15	0.82 (0.48–1.38)	0.45
Tertile 1 (<120)	24	18	1.00		1.00	
Tertile 2 (120–240)	31	17	0.84 (0.5–1.41)	0.51	0.60 (0.33–1.09)	0.09
Tertile 3 (>240)	20	20	0.65 (0.36–1.20)	0.17	0.49 (0.25–0.96)	0.04
*Cancer-specific survival^b^*						
Low (<median)	37	23	1.00		1.00	
High (≥median)	33	32	0.72 (0.45–1.15)	0.17	0.83 (0.48–1.42)	0.49
Tertile 1 (<120)	28	19	1.00		1.00	
Tertile 2 (120–240)	26	18	0.80 (0.47–1.37)	0.42	0.55 (0.29–1.04)	0.06
Tertile 3 (>240)	16	18	0.69 (0.37–1.27)	0.23	0.50 (0.26–0.99)	0.05

Abbreviations: CI = Confidence Intervals.

^a^Variables included in the adjusted analysis were age at diagnosis, gender, tumour nodal status, circumferential resection margin, tumour differentiation, lymphovascular invasion, smoking status and tumour location.

^b^This analysis included 125 patients as 5 had died due to other causes.

### Sensitivity analysis

Table 3 outlines sensitivity analysis restricted to junctional tumours. As with the main analysis, higher VDR expression was associated with improved overall survival and cancer-specific survival, and the magnitude of associations were strengthened. Patients with VDR expression in the highest tertile had a significant 56% reduction in all-cause mortality (HR 0.44 95% CI 0.22–0.99), with similar reductions in cancer-specific mortality, although statistical significance was slightly attenuated (HR 0.47 95% CI 0.21–1.02) compared to patients with the lowest VDR expression tumour cores.

**Table 3:** Sensitivity analysis of oesophageal adenocarcinoma survival outcomes according to Vitamin D receptor expression, restricted to junctional tumours only

	Dead *n* = 67	Alive *n* = 43	Adjusted Hazard ratio^a^ (95% CI)	*P*-value
*Overall survival*				
Tertile 1 (<120)	25	17	1.00	
Tertile 2 (120–240)	26	15	0.50 (0.26–0.97)	0.04
Tertile 3 (>240)	16	11	0.46 (0.21–0.98)	0.04
*Cancer-specific survival^b^*				
Tertile 1 (<120)	24	17	1.00	
Tertile 2 (120–240)	23	15	0.44 (0.21–0.89)	0.02
Tertile 3 (>240)	15	11	0.47 (0.22–1.02)	0.05

Abbreviations: CI = Confidence Intervals.

^a^Variables included in the adjusted analysis were age at diagnosis, gender, tumour nodal status, circumferential resection margin, tumour differentiation, lymphovascular invasion, and smoking status.

^b^This analysis included 105 patients as opposed to 110 patients as in the overall survival analysis as 5 had died due to other causes.

Secondary analysis in which all above outcomes were evaluated for median, rather than maximum, VDR expression largely showed similar results that were attenuated in statistical significance (data not shown).

## DISCUSSION

This study is the first to demonstrate a significant, dose-response, association between higher VDR expression and improved survival in patients with oesophageal adenocarcinoma. Sensitivity analysis also demonstrated that the association between higher VDR expression and improved survival was particularly evident in patients with oesophago-gastric junctional tumours.

These findings contrast with those from the only other published study to have considered the impact of VDR expression on oesophageal cancer survival. In a study of 116 patients in the USA, no significant difference in overall survival (21 months in high VDR expression versus 20 months in low VDR expression) was demonstrated between groups with high and low VDR expression (*p* = 0.99) [14]. There are multiple differences in that study's design compared with ours that may account for the conflicting results. Firstly, in our study, all patients underwent neoadjuvant treatment prior to surgical resection, whilst in the aforementioned study all patients had surgical resection without neo-adjuvant therapy [14]. Limited research is available to explore the impact of neoadjuvant treatment upon VDR expression of the primary tumour, although one small study in 15 patients found those with higher VDR expression were less likely to respond to treatment, indicating a potential interaction [16]. However, the authors accepted that these results could be simply due to chance [16]. Secondly, the methods of scoring and analysing the VDR expression differed in that there was only one sample taken for each patient and there was considered to be a high expression of VDR if 10% or more of cells stained with an intensity score of 2+ or 3+ [14]. This would be the equivalent of an H-score of 20 or 30 in our study to divide patients into high and low expression, whereas our lowest tertile reflected an H-score of less than 120. Our methods also involved scoring three cores rather than one from each tumour specimen, which reduces the likelihood of sampling bias. Thirdly, there may be underlying differences in the population studied (Northern Ireland and the USA) and fourthly, this study used a different antibody to stain the specimens. All of these factors may account somewhat for the difference in findings.

Although there is limited research looking at the impact of high VDR expression in oesophageal adenocarcinoma outcomes, there are multiple other clinical studies which look at the impact of high VDR expression on survival in patients with other cancer sites. Some, but not all, studies within colorectal cancer patients have demonstrated that higher VDR expression is associated with improved survival [8, 17, 18]. A single study looking at the impact of VDR expression on survival in pancreatic adenocarcinoma performed in a Chinese population of 61 patients found that patients with high VDR expression survived longer than those with low or no VDR expression [9]. Similar results were found in a single study looking at the impact of VDR expression in cholangiocarcinoma in a Thai study of 111 patients. Patients with no VDR expression had a 2-fold higher increased risk of death than in patients whose tumours expressed VDR (HR 2.00 95% CI 1.07–3.76) [19]. Studies in melanoma skin cancer, breast cancer, oesophageal squamous cell carcinoma and urothelial bladder cancer have also found similar findings with improved overall survival and progression free survival in patients with higher VDR expression within the tumour tissue [11–12].

Another interesting feature of previous studies in other cancer sites was that higher VDR expression was more frequently observed in well differentiated tumours compared to poorly differentiated tumours in colorectal cancer, pancreatic adenocarcinoma and cholangiocarcinoma [8, 9, 19]. This corroborates similar findings in a previous study by Trowbridge *et al.* in tissue from 15 oesophageal adenocarcinoma patients [16]. In our study however, there was no association between tumour grade and VDR expression, and adjustment for grade within our survival analysis did not affect our results.

When we performed sensitivity analysis for junctional tumours only, there was a greater magnitude of association between VDR expression and overall survival and disease-specific survival, although statistical significance was slightly attenuated for the latter. Previous findings suggest that VDR expression only features in oesophageal cells after they have undergone metaplastic transition, but it is unclear if this is a cause-or-effect role [15, 20]. Our findings in the sensitivity analysis may therefore arise due to a difference in VDR expression between tumour sites. This theory is supported by the fact that junctional tumours can arise from oesophageal tissue or gastric tissue and these tissues may differ biologically in both their normal state and pre-malignant state.

Evidence from our study demonstrates for the first time that any association between circulating vitamin D levels and oesophageal adenocarcinoma outcomes, mediated by VDR expression, may be biologically plausible. VDR are activated when they combine with vitamin D_3_, the active form of vitamin D. This subsequently combines with the retinoid x receptor which can then promote or suppress hallmarks of tumorigenesis [5, 6]. Indeed, a cell-line study has demonstrated that VDR activation regulates apoptosis and cell differentiation, and suppresses tumour proliferation [4]. However, further studies that incorporate assessment of serum 25-hydroxyvitamin D status in addition to tumour VDR expression in patients with oesophageal adenocarcinoma are required to corroborate this hypothesis.

Research into the impact of vitamin D with respect to oesophageal adenocarcinoma risk is highly controversial, with observational studies having reported null results, decreased risks, or indeed increased risks of oesophageal adenocarcinoma in patients with higher levels of vitamin D intake or status [21–24]. No clear associations for circulating vitamin D levels and prognosis after a diagnosis of oesophageal cancer were identified in a European cohort study, although that included only 74 adenocarcinoma patients [25]. Our study provides some evidence that VDR is associated with oesophageal adenocarcinoma outcomes, and therefore indirect evidence of a biological role for vitamin D.

One observational study has investigated the impact of post-operative vitamin D supplementation in patients having undergone surgical resection for their oesophageal squamous cell carcinoma [26]. There were 280 patients with oesophageal squamous cell carcinoma and of these there were 49 patients who took daily vitamin D supplements of between 200–400 international units daily [26]. Although vitamin D supplement use was not associated with improved overall survival, it was associated with a 39% reduction in disease recurrence in adjusted analysis (HR 0.61 95% CI 0.38–0.98) [26]. This demonstrates the role that vitamin D may play in the post-operative phase in oesophageal squamous cell carcinoma.

Theoretically, if VDR levels impact on survival and vitamin D activates the VDR, then vitamin D may play a role in the neo-adjuvant treatment phase or even in the chemo-preventative setting [3, 27]. This hypothesis is supported by findings in other cancers with *in-vivo* studies in colorectal, pancreatic and gastric cancer having shown 1,25-dihydroxyvitamin D_3,_ 25-hydroxyvitamin D_3_ and vitamin D analogues to decrease cancer cell growth [28–30]. Furthermore, results in animal models have been encouraging as vitamin D3 has been shown to decrease both pre-cancerous and cancerous lesions in gastric cancer in rats [31].

This study has several strengths, including being the first study to identify VDR as potential biomarker to predict outcomes in oesophageal adenocarcinoma, and only the second study to investigate this association. Furthermore, our study was performed in a population which has low exposure to vitamin D and a high incidence of oesophageal adenocarcinoma [2, 32]. Survival remains poor in this disease with limited treatment options and this study enables hypothesis generation around the role of vitamin D analogues in the management of oesophageal adenocarcinoma [33].

The main limitation of this study is the relatively small sample size, although our cohort size of 130 patients is typical of this relatively rare disease site. All patients had surgically resectable disease, therefore this cohort represents patients with more favourable prognosis, and we cannot deduce if VDR expression impacts upon the outcome in patients with more advanced disease. Future studies should aim to evaluate associations between VDR expression and survival in patients with advanced disease as has been demonstrated for other cancer sites, however given the limited expected survival within this group of patients it may be difficult to detect significant benefits [34].

In conclusion, in this Northern Irish population, patients with higher VDR expression in oesophageal adenocarcinoma have a more favourable prognosis. However, further work is needed to validate these findings and define the role of VDR in the aetiology, management and progression of oesophageal adenocarcinoma.

## MATERIALS AND METHODS

This study was performed and reported in line with the REMARK guidelines [35].

### Patient selection

**Figure 1:**
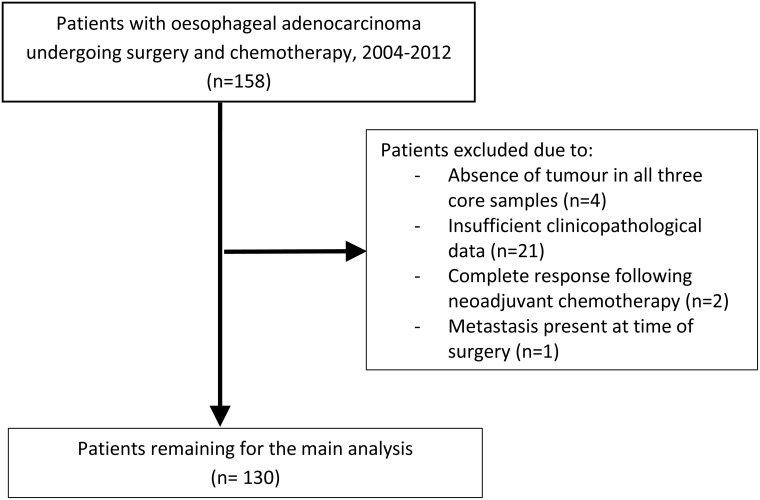
Flow chart demonstrating reasons for patient exclusion from study

In this population-representative study, all patients in Northern Ireland who underwent neoadjuvant chemotherapy followed by surgical resection for oesophageal adenocarcinoma between the 1st January 2004 and the 31st December 2012 were included. There were 158 formalin-fixed paraffin embedded (FFPE) oesophageal adenocarcinoma resection specimens collected from the Northern Ireland Cancer Centre. Of these, slides with tumour which could be scored and matched clinical information was available for 137 patients. Seven patients were excluded from further analysis, as two were staged as T0 disease, one had metastatic disease, and tissue microarray (TMA) cores were unable to be scored for VDR expression for four patients, leaving 130 patients for inclusion in our final analysis. Figure 1 summarises the indications for exclusion from this study.

### Clinical data

Clinical data and information on study outcomes up until 31st December 2014 was retrieved via patient note review at the Northern Ireland Cancer Centre. Information included age at diagnosis, date of diagnosis, date of surgery, patient sex, smoking and alcohol status. Pathology reports were reviewed for tumour characteristics including tumour location, presence of lymphovascular invasion, circumferential resection margin status, tumour differentiation and TNM stage. Tumour location was divided into lower third of oesophagus (greater than 5 cm proximal to the oesophagogastric junction), Siewert 1 (within 1–5 cm above the oesophagogastric junction), Siewert 2 (within 1 cm above and 2 cm below the oesophagogastric junction) and Siewert 3 (2–5 cm below the oesophagogastric junction) [36]. Pathological staging was defined according to International Union Against Cancer (UICC) TNM staging, 7th edition [37]. Finally, the date and cause of death were recorded, where applicable.

### Construction of tissue microarrays

A FFPE tissue block was selected from each resection specimen and three 1mm cores of tumour were embedded in a paraffin block using the Beecher Manual Arrayer^®^.

### Immunohistochemistry staining and scoring

Immunohistochemical analysis was performed within the NI-Molecular Pathology Laboratory (NI-MPL) at Queen's University Belfast, following approval by the Northern Ireland Biobank (study number NIB15-0176). Slides were immunostained on a Ventana BenchMark fully automated immunostainer, with a previously validated rabbit monoclonal VDR antibody (cell signalling-clone number D2K6W: 1/100, pre-treatment CC1 32 minutes, Optiview detection without amplification) to enable VDR expression to be identified and then scanned on an Aperio AT2 scanner, and viewed as digital images on Xplore (PathXL).

**Figure 2:**
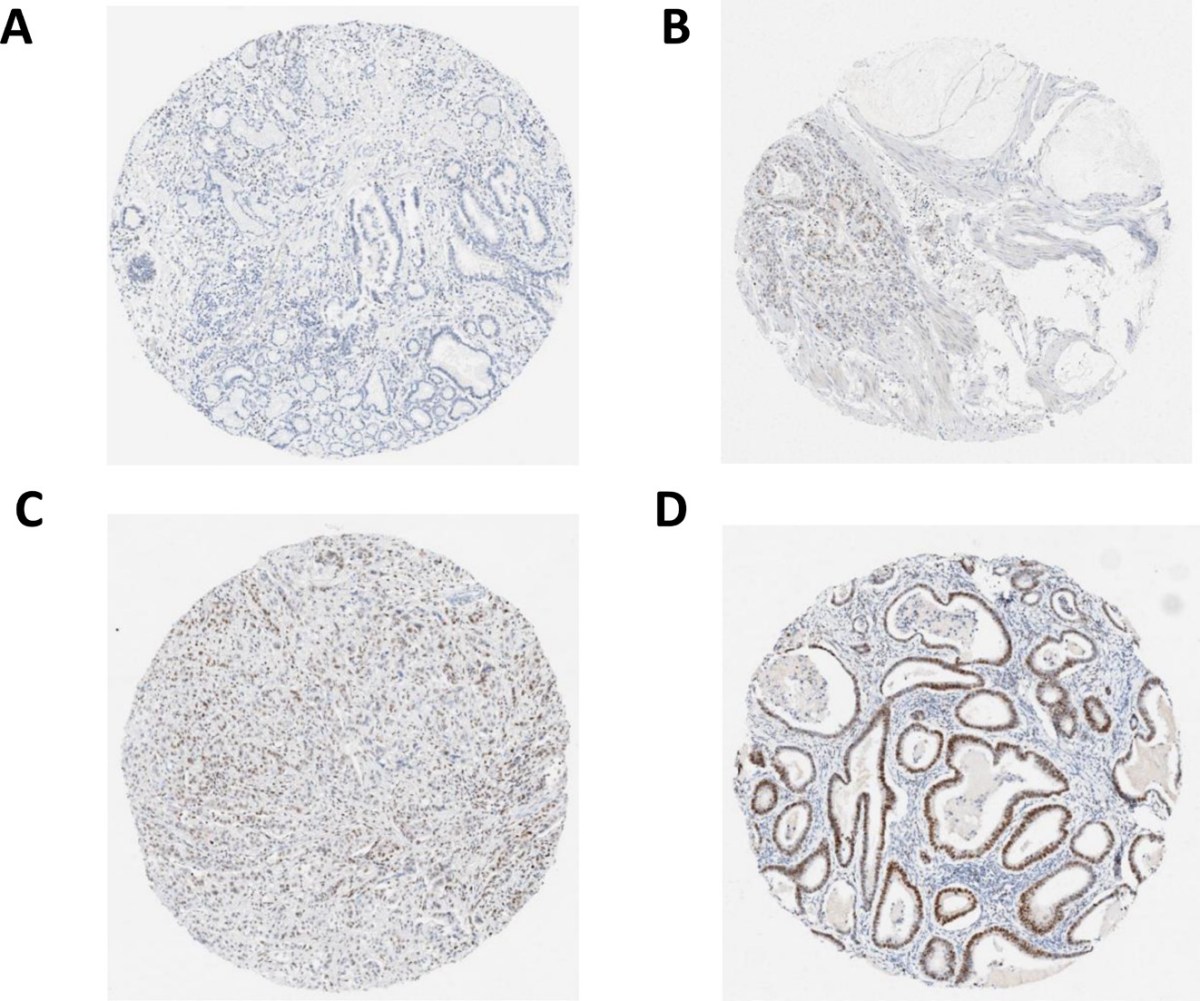
(**A**) no staining [intensity 0]; (**B**) weak staining [intensity 1]; (**C**) moderate staining [intensity 2]; (**D**) strong staining [intensity 3].

Biomarker expression was evaluated by a trainee pathologist (JT) and an independent observer (surgical registrar, SMcC), who were both blinded to clinical data. This process was carried out following the training and guidance of an expert gastrointestinal pathologist (DMcM). The staining intensity from each tissue section was assessed along with the percentage of tumour cells staining positive and a final agreement on discordant results was made. Scoring was based on intensity (0 = no staining, 1 = weak, 2 = moderate and 3 = strong staining observed) and this was multiplied with the percentage of tumour cells staining positive to give an H-score between 0 and 300. Examples of these different grades of staining are demonstrated in Figure 2. Different methods for evaluating the VDR expression are reported in the literature and may contribute to different results. A range of these methods are displayed in Table 4.

**Table 4:** Different methods of calculating the VDR expression in other studies

Study author	Type of cancer	Method of staining for VDR	Method of scoring grade of staining	Method of scoring percentage of cells staining positive	Description of how the overall score and cut off points were calculated
Our study	Oesophageal adenocarcinoma	Rabbit monoclonal VDR antibody	0-no staining 1-Weak 2-Moderate 3-Strong	Percentage of tumour cells staining positive	Grade multiplied by percentage to give H-score between 0–300
Zhou *et al.* [14]	Oesophageal adenocarcinoma	Mouse mono-clonal anti-VDR antibody	0-no staining 1+Weak 2+Moderate 3+Strong	>or< than 10% of tumour cells staining positive	If 10% or more of cells stained 2+ or 3+ was deemed to have high expression
Wang *et al.* [9]	Pancreatic adenocarcinoma	3,3 diaminobenzidine with western blot	0-no staining 1-Weak 2-Moderate 3-Strong	Score 0 (<5%) Score 1 (6–25%) Score 2 (26–50%) Score 3 (51–75%) Score 4 (76–100%)	Grade score multiplied by percentage score. Maximum of 12 with <4 low expression and 4 or greater high expression
Seubwai *et al.* [22]	Cholangiocarcinoma	Rat anti-VDR monoclonal antibody	Did not score grade	0 = negative 1+ (1%–25%) 2+ (26%–50%) 3+ (>50%)	Patients were grouped into one of the four groups as per the percentage of staining. Analysis often involved presence versus absence of VDR
Ditsch *et al.* [10]	Breast Cancer	Vitamin D antibody (monoclonal clone 2F4 isotype IgG2a, AbD Serotec)	0-no staining 1-Weak 2-Moderate 3-Strong	0 = negative 1 = <10% 2 = 11%–50% 3 = 51%–80% 4 =>81%	Grade was multiplied by percenatage that stained positive with a maximum score of 12. 0–1 indicated no staining, 2–4 was moderate staining and 6–12 was high staining
Brozna *et al.* [11]	Cutaneous melanoma	Monoclonal antibodies (clone 97A, Abcam Inc, Cambridge)	0-no staining 1-Weak 2-Moderate 3-Strong	Did not score percentage	Cut off points were as per the three levels of grading

### Statistical analysis

Median and maximum H-scores for VDR expression across the triplicate tumour cores were generated for each patient, to explore if there was an association with outcomes. Following evaluation of histograms, the median and maximum H-scores were used to create groupings of both high/low VDR expression and tertiles of high/medium/low VDR expression, although the distributions in the histograms did appear to favour the latter method. Primary analysis evaluated the distribution based on the maximum H-score (which may be more clinically relevant) from the triplicate cores in TMAs, and median H-scores (which may be less subject to sampling errors) were evaluated in secondary analysis.

Patient demographics and tumour characteristics according to tumour VDR status were compared using chi-squared tests. Outcomes studied included the impact of VDR expression across tertiles on overall survival (death from any cause) and cancer-specific survival (death from oesophageal adenocarcinoma). Comparison between VDR status and prognosis was evaluated using Cox proportional hazards regression models for unadjusted and adjusted results. The variables included in the adjusted analysis were age at diagnosis, gender, tumour nodal status, circumferential resection margin, tumour differentiation, lymphovascular invasion, smoking status and tumour location. Sensitivity analysis was performed for junctional tumours separately. Stata version 14.2 (College Station, TX, USA) was used for statistical analysis.

## Abbreviations

VDR: Vitamin D receptor
